# Task-Evoked Dynamic Network Analysis Through Hidden Markov Modeling

**DOI:** 10.3389/fnins.2018.00603

**Published:** 2018-08-28

**Authors:** Andrew J. Quinn, Diego Vidaurre, Romesh Abeysuriya, Robert Becker, Anna C. Nobre, Mark W. Woolrich

**Affiliations:** ^1^Oxford Centre for Human Brain Activity, University of Oxford, Oxford, United Kingdom; ^2^Wellcome Centre for Integrative Neuroimaging, University of Oxford, Oxford, United Kingdom; ^3^Department of Experimental Psychology, University of Oxford, Oxford, United Kingdom

**Keywords:** magnetoencephalography, MEG analysis, network, dynamic, hidden Markov model

## Abstract

Complex thought and behavior arise through dynamic recruitment of large-scale brain networks. The signatures of this process may be observable in electrophysiological data; yet robust modeling of rapidly changing functional network structure on rapid cognitive timescales remains a considerable challenge. Here, we present one potential solution using Hidden Markov Models (HMMs), which are able to identify brain states characterized by engaging distinct functional networks that reoccur over time. We show how the HMM can be inferred on continuous, parcellated source-space Magnetoencephalography (MEG) task data in an unsupervised manner, without any knowledge of the task timings. We apply this to a freely available MEG dataset in which participants completed a face perception task, and reveal task-dependent HMM states that represent whole-brain dynamic networks transiently bursting at millisecond time scales as cognition unfolds. The analysis pipeline demonstrates a general way in which the HMM can be used to do a statistically valid whole-brain, group-level task analysis on MEG task data, which could be readily adapted to a wide range of task-based studies.

## Introduction

It is likely that the brain supports complex thought and behavior by dynamic recruitment of whole-brain networks across millisecond time-scales. The signatures of these dynamics may be observable in M/EEG data, although robust modeling of the evolution of functional connectivity on rapid cognitive timescales remains a challenge (O’Neill et al., 2017a). Network dynamics are observable using sliding time-window approaches on both resting state (de Pasquale et al., 2010, 2012) and task data (O’Neill et al., 2017b). Yet the temporal resolution of sliding window approaches is limited, as each window requires relatively large amounts of data, typically several seconds in length. In particular, a short window length will give poor estimation of the graphical networks, while a long window length limits the visibility of fast time-scale in the sliding window analysis.

Alternatively, Hidden Markov Modeling can be used to segment observed data into a set of discrete functional states that reoccur over time. Hidden Markov Models (HMMs) do not require pre-specification of sliding window length, instead the relevant time-scales are learnt directly form the data. The HMM estimates adaptive state visits or ‘windows’ in a data driven manner. State-wise functional connectivity can be robustly estimated by pooling data across all visits to each state, though each individual visit may only last for tens or hundreds of milliseconds. The result is that the HMM is capable of identifying dynamic re-organization of whole brain networks on fast, milliseconds time-scales. Previous applications of HMMs to source MEG data have shown state switching between large-scale networks on the order of 100 ms (Baker et al., 2014).

The HMM can be used to flexibly characterize dynamic states across a range of data modalities and has been applied to an increasing number of tasks and datasets. This includes time-varying oscillations during finger tapping in source reconstructed MEG signals (Vidaurre et al., 2016), identifying processing stages during cognitive tasks in EEG (Borst and Anderson, 2015), and finding state sequences associated with perception and recall of narrative structure in fMRI (Baldassano et al., 2017). These examples show the ability of the HMM to represent behaviourally relevant dynamics within its states and state time-courses. This approach can be extended to explore the relationship between states and cognition in very large datasets using Stochastic Inference (Vidaurre et al., 2017a). For example, HMM states inferred on 820 fMRI datasets from the Human Connectome Project revealed a hierarchical temporal structure, where the switching and rate of occurrence of brain states was shown to be both heritable and predicative of psychological traits (Vidaurre et al., 2017c).

Here we present a group-level HMM analysis of source-space MEG data during a face processing task collected by (Wakeman and Henson, 2015). This demonstrates a general way in which the HMM can be used to do a statistically valid whole-brain, group-level task analysis on MEG task data, which could be readily adapted to a wide-range of task studies. This approach reveals task dependent whole-brain dynamics at millisecond time-scales as cognitive processes unfold. Two different HMM models are fitted to the data. Firstly we use an HMM on the broadband power envelope of source MEG signals, which is able to identify states with distinct networks of power, similar to the approach used on resting state MEG data in (Baker et al., 2014). Secondly, we use a time-delay embedded HMM on the raw source MEG signals, which is able to identify states with distinct multi-region spectral properties and phase locking networks, similar to the approach used on resting state MEG data in (Vidaurre et al., 2017b). The intention is not to statistically compare these two approaches but to provide some insight into their use and the information they provide. These two HMM variants provide an alternative representation of frequency domain task-responses as fast transient events of distinct multi-region spectral patterns. Each event on a single trial may only burst for tens of milliseconds, but following averaging across many trials can lead to an apparently sustained response in a similar manner to the work in (Shin et al., 2017).

We demonstrate the use of the HMM for the analysis of task data on a freely available MEG dataset in which subjects are viewing Face or Scrambled Face stimuli, while making a subjective decision about the symmetry of the image and responding with a button press (Wakeman and Henson, 2015). Completion of this task is expected to recruit visual perception, decision-making and motor processes in rapid succession over the course of a trial (∼1.5 s). The HMM provides a means to interrogate the dynamic recruitment of networks as these processes unfold within the brain on fast sub-second time-scales.

## Materials and Methods

### Hidden Markov Models

Here, we summarize the Hidden Markov Model and its use for describing source-space MEG data. A detailed introduction to the general theory of Hidden Markov Modeling can be found in (Rabiner and Juang, 1986) whilst more detail on the specific implementation for MEG data can be found in (Rezek and Roberts, 2005; Baker et al., 2014; Vidaurre et al., 2016, 2017a)

**FIGURE 1:**
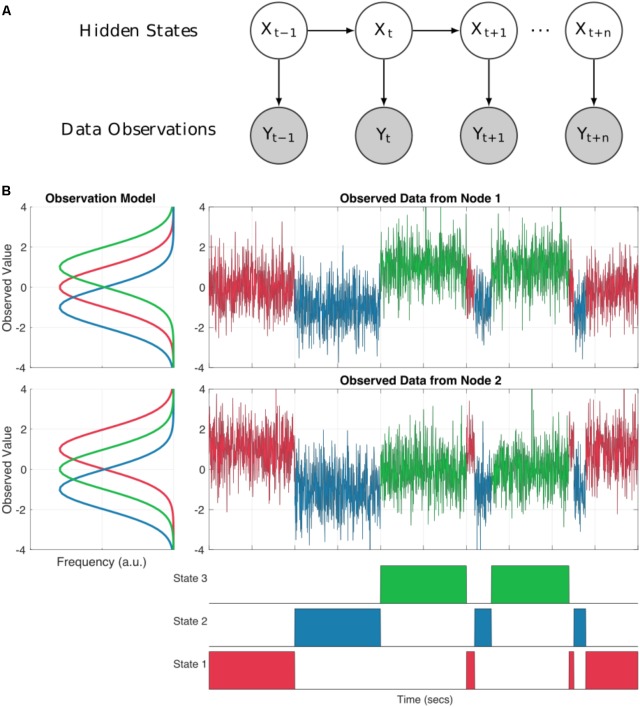
**(A)** A schematic of a Hidden Markov Model. Each sample at a given time point t is described as one of a set of discrete hidden states denoted by Xt. Each state has an observation model Y which characterized the distributions of the observed data whilst state X is ‘on’. **(B)** An illustration of a two node system moving through three HMM states. The observation model for each node is shown in the line plots to the left. Each state has a distribution for each node describing the observed values whilst that state is ‘on’. The time-series to the top right show the observed data for each node. The values are color-coded according to which state is on at each time point. Note that at each point in time the observed values are drawn from the distribution of the appropriate observation for that sample. The bottom row shows the true state time-course for the system.

A Hidden Markov Model (HMM) can represent dynamics in the brain as a system moving through a set of discrete states. The states are mutually exclusive, in that only one may occur at any one point in time (although this assumption is relaxed by the use of soft, probabilistic inference) and Markovian, in that the next state is only dependent on the current state. Critically, the states are abstract (hidden) and not directly observable from the data. The link between these hidden states and our observed data comes from an observation model (also known as *emission probabilities* or *output probabilities*). Each state has its own observation model defining a probability distribution from which our observed data is drawn whilst our system is in that state (see **Figure 1A**). This separation between the underlying state dynamics and the form of the observation models make the HMM a highly flexible framework. We may tune the observation model to suit a range of modalities or datasets whilst keeping the HMM inference and wider statistical framework constant.

In neuroimaging applications, the HMM observation model can be set up to match existing approaches in static functional connectivity estimation. For instance, a Gaussian Graphical Model can be estimated to provide a probabilistic description of functional brain data with a joint-multivariate normal distribution across brain regions. Such models are completely characterized by their covariance matrix (Bishop, 2006), which describes the functional connectivity within the network. This approach has been widely adopted for static functional-connectivity estimation in fMRI (Marrelec et al., 2006; Varoquaux et al., 2010) and MEG (Colclough et al., 2017). The HMM provides a temporal extension to this approach by tuning the observation model of each state to describe a distinct multivariate-normal distribution. In other words, each state’s observation model takes the same distributional form as a static functional connectivity estimate. The switching between states then describes switching between large-scale undirected Gaussian Graphical Models, each containing a description of the functional activity within the network (Baker et al., 2014). The observation model may be further tuned depending upon the specific needs of a dataset or modality, making the HMM a highly flexible framework. For instance, (Vidaurre et al., 2016) observes a multivariate autoregressive model describing the raw MEG time-courses.

When looking to characterize the spatial and temporal properties of the brain, the HMM uses a full network graph across N nodes, i.e., a [N × N] multivariate Gaussian process is specified at each time-point. The dynamics of this network over time (T) is described by the transitions between states in the state-time course. In contrast, models such as ICA (Brookes et al., 2011) or micro-states (Lehmann and Skrandies, 1984; Koenig et al., 2005) seek to decompose neural data into sets of activation patterns and their temporal evolution. The spatial component of these models are often referred to as “networks” despite the fact that they are not full network graphs and correspond to spatial maps defined by [N × 1] vectors; this is in contrast to the full [N × N] graphical networks used in the HMM.

As an illustration, **Figure 1B** shows how the HMM can be used to describe a simulated bivariate time-series. Here the observed data are generated using known Gaussian observation models and state time-courses from three states. For real data analysis, we would start with the raw data and the inference would estimate the parameters of the observation models and state time-courses. In our simulation, each states observation model generates data with different mean values for the two nodes summarized by the distributions on the left hand side of **Figure 1B**. The first state has a mean of zero in the first time-series and a mean of one in the second. The second state has a mean of -1 in both nodes and the third state has a mean of one in times-series one, and zero in time-series two. The observed data at each time point is randomly drawn from the distribution of the currently active state as defined by the state time-course.

Formally, the HMM requires us to pre-specify the number of hidden states (K) and the form of the observation models. The number of states (K) is important to explore when fitting a HMM to a new dataset. This is to confirm that the number of states is sufficient to provide a useful description of the dataset whilst ensuring that key results are robust to changing the number of states inferred. A number of approaches may be useful in exploring this. Firstly the final Free Energy in the HMM inference is an approximation to the model evidence and may be used to formally compare models. If we explore a set of HMMs with different values of K, we would prefer to take the model with the lowest value of free energy as this represents the model which best fits the data with the fewest parameters. In practice, the Free Energy is likely to monotonically decrease with increasing values of K making objective choice of the ‘best’ model difficult. A more subjective approach is to ensure that the results are valid across HMMs with different numbers of states. The HMM may be estimated with different values of K and compared. This approach is used in (Baker et al., 2014, **Figure 2**-figure supplement 1) to show that changing K did not change the topographies of the most prominent states. Finally, the inference in the HMM-MAR toolbox is adaptive to some extent, such that if K is higher than is supported by the data, then the variational inference scheme can prune out the excess of states. From a purely quantitative point of view, a more optimal estimation of the number of states would require the use of the so-called infinite Hidden Markov model (Beal et al., 2002), which is however less practical to apply on electrophysiological data due to its high computational cost (Nielsen et al., 2017).

**FIGURE 2:**
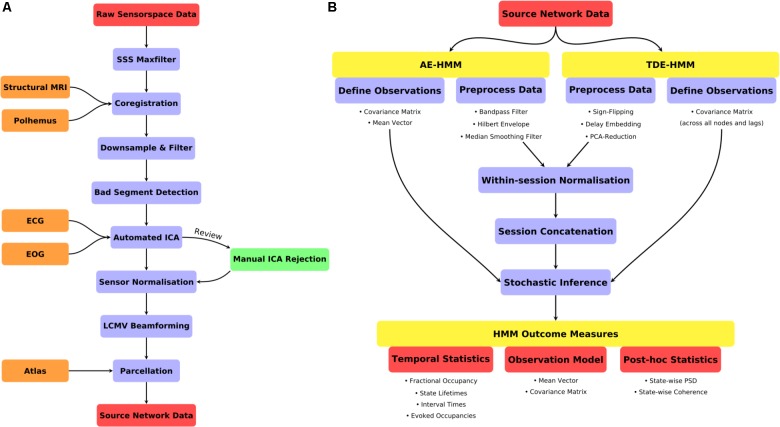
**(A)** A schematic for the preprocessing pipeline used prior to HMM analysis. **(B)** An illustration of the HMM definition, preprocessing and post-stats.

### Software

All analyses are performed using freely available tools in MATLAB. The code carrying out the analysis in this paper can be found here: https://github.com/OHBA-analysis/Quinn2018_TaskHMM. This analysis depends on a number of other toolboxes and software packages. The preprocessing and source-space parcellation analyses are performed using the OHBA Software Library (OSL^1^). This builds upon Fieldtrip, SPM and FSL to provide a range of useful tools for M/EEG analyses. The HMM is inferred using the HMM-MAR toolbox^2^. All the software and scripts to carry out the analyses can be downloaded from the project hosted on the Open Science Framework (Quinn et al., 2018^3^). The HMM analyses can be very computationally intensive even on a modern computer system. The analyses in this paper were computed on a Linux workstation with an Intel Xeon E5 CPU clocked at 1.90 GHz and 32 Gb of RAM. The analysis can be adapted to work on computers with less RAM by changing the Stochastic Inference Batch settings in the Stochastic Inference section.

Details on installation and setup of the dependencies can be found in the README.md file in the main study repository. In brief, the OSL and HMM-MAR toolboxes must be on the MATLAB path and initialised. Please note, that all file paths specified here are relative to the location of the download accompanying this paper, ie the paths assume that the top-level of the download is your present working directory. Firstly, the paths in scripts/+utils/get_studyinfo.m should be edited to specify the location of the downloaded toolboxes and data as well as the directory to save generated analyses into. utils.get_studyinfo returns a config structure storing these paths in and is routinely used within the other analysis scripts. Finally, the path to the downloaded scripts should be added add the top of scripts/hmm_0_initialise.m. Running this script adds all the relevant paths and toolboxes to the MATLAB path ready for subsequent analyses. The analyses depend on the use of SPM12 and FSL 5.0.9 on a unix-type system.

The following sections describe the pre-processing stages performed prior to Hidden Markov Modeling. These steps process the continuous data after Maxfilter processing through to source-space parcellated time-series. The descriptions are annotated with specific function calls where appropriate. More details can be found in the accompanying script hmm_1_prepreocessing.m in the “scripts” directory.

### Data Acquisition and Experimental Design

Analysis was carried out on MEG data (acquired on a Elekta Neuromag Vectorview 306) in a freely available dataset in which 19 participants completed a simple visual perception task using pictures of faces (Wakeman and Henson, 2015^4^; Revision 0.1.1). Each participant completed six scans in which they viewed sets of famous, unfamiliar or scrambled faces whilst making a perceptual judgment on the symmetry of the faces. Each trial begins with a fixation cross onset between 400 and 600 ms before a target stimulus appears. The target is either the face or scrambled face stimulus, and remains onscreen for between 800 and 1000 ms. Further details of the data acquisition and experimental design can be found in (Wakeman and Henson, 2015).

### Data Preprocessing

A summary of the data preprocessing pipeline can be seen in **Figure 2A**. All the code for the preprocessing can be found in script hmm_1_preprocessing.m.

#### Maxfilter

The analysis starts with the MEG and structural MRI data downloaded from Revision 0.1.1 of (Wakeman and Henson, 2015)^4^. The online dataset includes both raw MEG datasets (eg., run_01_raw.fif) and MEG datasets that have undergone Maxfilter processing (eg., run_01_sss.fif). Maxfilter is a method for separating which parts of the recorded MEG signal arise from neuronal activity within the brain, and which come from external noise sources. In the interest of reproducibility, our analysis begins with the continuous MEG data that have already been processed with Maxfilter as described in (Wakeman and Henson, 2015).

#### Data Import

The first stage of hmm_1_preprocessing.m runs a short check to ensure that the maxfilter preprocessed data can be found in the location specified in the get_studyinfo datadir variable. The data are then converted to SPM12 format and copied into a spm_sss directory within the specified analysis directory. The import is performed using osl_import.m and the copy with spm_eeg_copy.m.

#### Coregistration

Registration between structural MRI and the MEG data was carried out using RHINO (Registration of Headshapes Including Nose in OSL). This uses scalp extraction tools in FSL and is designed to make full use of Polhemus head shape points, including those on the nose, during coregistration. In this case, the data provided by (Wakeman and Henson, 2015) has been de-faced to ensure participant anonymity in this publicly available dataset. As such, here we perform the coregistration using only the Fiducial landmarks, but generally recommend using a large number of Polhemous headshape points across the scalp, forehead and nose to ensure a high quality registration. The coregistration is performed using osl_headmodel.m.

The importing and coregistration steps process file structure and meta-data, and do not impact the MEG data itself. The following analysis stages all involve manipulation and denoising of the MEG data within the SPM12 objects in the spm_sss directory.

#### Downsampling and Filtering

The MEG data were down sampled to 250 Hz to reduce computational demands and the amount of disk space consumed by the analyses. Secondly, a band pass filter was applied from 1–45 Hz, to remove very slow trends and high frequencies in the dataset.

These steps are implemented using spm_eeg_downsample.m and osl_filter.m (a wrapper around spm_eeg_filter.m).

#### Bad Segment Detection

Time segments containing artifacts were detected using an automatic algorithm to ensure reproducibility and avoid user bias that may be introduced by manual artifact detection. Bad segments were rejected by identifying outliers in the standard deviation of the signal computed across all sensors in 1s non-overlapping windows. Outliers were identified using the generalized extreme Studentized deviate method (Rosner, 1983) at a significance level of a 0.05 and with the maximum number of outliers limited to 20% of the data set. The windows corresponding to the outliers were then marked as bad samples in the continuous dataset and excluded from subsequent preprocessing and analysis. The bad segment detection is performed using osl_detect_artifacts.m which returns an SPM object with any identified bad segments marked as artifact events.

#### Independent Component Analysis

Further de-noising was applied using temporal Independent Components Analysis (ICA) across the sensors using the FastICA algorithm (Hyvarinen, 1999) on only the ‘good’ time-samples remaining after Bad Segment Detection, and was run separately on each session of data. ICA separates a signal into a set of additive non-Gaussian subcomponents that are statistically independent from one another. These components may be inspected to identify components that describe ‘noise’ sources in the data. These noise components may then be removed from the analysis. Importantly, each Independent Component is the length of the whole recording and as such its removal will have some effect on every sample. Therefore, if a component identifies a short-lived artifact, its subtraction will still have an affect on the rest of the dataset. Given this, we use ICA to identify artifacts that reoccur regularly throughout the entire dataset such as eye movement and the heartbeat. Any short-lived artifacts or periods of high variance should be removed in the bad segment detection stage. If the ICA yields components with large brief artifacts, we recommend returning to the bad segment detection and ensuring that the duration of the artifact is marked as bad, before re-estimating the ICA on the remaining data.

The ICA components were correlated with the Electrooculogram (EOG) and Electrocardiogram (ECG) artifact channels to identify likely ‘noise’ components. Any components with a correlation greater than *r* = 0.5 were removed by subtraction of those spatio-temporal components from the decomposition. The results were checked by hand if: a) the session had zero or greater than four noise components, or b) if no candidate noise component was found for the EOG or ECG. On average, 2–3 components were rejected across the 114 scan sessions. The ICA is performed using osl_africa.m which performs the ICA estimation, automatic bad component detection and reconstruction of the data from the good components. This returns an SPM object with the ICA reconstructed data included as an online-montage.

#### Sensor Normalization

The Elekta Neuromag system contains both Magnetometers and Planar-Gradiometers. These sensor-types have variances on different orders of magnitude and so do not equally contribute to the covariance matrix calculation during beamformer estimation. To reduce this disparity, the two sensor-types were normalized prior to beamforming. An eigenvalue decomposition was computed across sensors within each coil type, and the data divided by the smallest eigenvalue within each (Woolrich et al., 2011). This was carried out separately on each session of data. This is performed using the normalize_sensor_data.m function within OSL.

#### Source Localisation

The continuous sensor data were projected onto an 8 mm grid in source space using a Linearly Constrained Minimum Variance (LCMV) vector beamformer (Veen and Buckley, 1988; Woolrich et al., 2011) carried out separately on each session of data.

The beamformer weights were estimated across an 8 mm grid cast within the inner-skull of the MNI152 brain. Defining the grid in MNI space ensures that we have the same number of grid points in each dataset and that the location of these points is comparable across participants. This is particularly relevant when applying the parcellation in the next section.

A covariance matrix was computed across the whole time-course and was regularized to 50 dimensions using PCA rank reduction. Note that Maxfilter reduces the dimensionality of MEG data to ∼64. Regularizing the covariance estimation to 50 dimensions removes the influence of the smaller components in the dataset and preserves the contribution from the largest components. A reduction to 50 dimensions was chosen as the SSS Maxfilter reduces dimensionality to ∼64, which ICA further reduces to ∼62 (an average of two ICA components were removed per dataset). A rank of 50 is conservatively below this upper limit on the dimensionality. The source localisation is performed using osl_inverse_model.m.

#### Parcellation and Leakage Reduction

Parcel-wise time courses were estimated and orthogonalised following the methods in (Colclough et al., 2015, 2016). A weighted (non-binary) parcellation with 39 cortical regions was applied. A single time-course was estimated per node from the first principle component across voxels, with voxel contributions weighted by the parcellation. Parcellation is performed using the ROInets.get_node_tcs function within the ROInets module of OSL.

Spatial leakage is a major confound when considering network connectivity estimates in MEG source space. This arises from the blurring of sources from their true locations into neighboring regions. To attenuate these effects, symmetric multivariate leakage correction was applied across the whole network (Colclough et al., 2015). This is a multivariate extension of previous orthogonalisation methods that identifies the set of orthogonal time-courses that are least displaced from the original, unmodified time-series. This is a conservative approach that removes all zero-lag correlation from the dataset, and could potentially be removing true neuronal functional connections at, or close to, zero-lag. One interesting alternative is the “innovations” approach, which looks to remove only the spurious zero-lag interactions, by effectively estimating the required orthogonalisation on the residuals of a multivariate autoregressive processes (Pascual-Marqui et al., 2017). However, this does require the choice of an appropriate model order for the multivariate autoregressive process; and in practice on real MEG data we have observed little difference between the “innovations” and “symmetric” leakage correction approaches. Another alternative is to build in models of the cross-talk functions into the spatial leakage correction (Hauk and Stenroos, 2014).

Orthogonalisation is performed in the first stage of hmm_2_envelope_estimation.m and hmm_3_embedded_ estimation.m as the two HMM variants are processed slightly differently immediately prior to inference.

#### Epoching

Epochs were defined based on the trigger channel in each session. Nine conditions were extracted. There are three repeat conditions (First, Immediate and Last) of each of three Face conditions (Famous, Unfamiliar and Scrambled). A trial structure containing the start and end samples for each epoch and the corresponding condition label was saved for each session for later use on the HMM state time-course. Crucially, the epoching was not applied to the MEG data used for the HMM inference, rather, the continuous MEG data is used without knowledge of the task structure or timings. For simplicity, HMM analyses use contrasts between the three Face conditions across all repeat conditions. Differences between the three repeat levels are not estimated in this analysis.

Epoching is performed using spm_eeg_definetrial.m within hmm_1_preprocessing.m.

### Hidden Markov Model Inference

Two variants of the HMM were estimated. Firstly, an Amplitude Envelope HMM (AE-HMM) was estimated to describe broadband power following the methods in (Baker et al., 2014). Secondly, we estimated a Time-Delay Embedded HMM (TDE-HMM) to characterize spectrally resolved networks characterized by Power-spectral densities and phase-locking. We did not apply the HMM with Multivariate Autoregressive observations (Vidaurre et al., 2016) due to the number of channels (parcels) in this data, it is only effective at modeling a smaller number of signals. Both the AE-HMM and TDE-HMM are appropriate for application to large-scale brain networks inferred from parcellated source-space MEG data. A summary of the HMM definition, inference and post-statistics is shown in **Figure 2**.

Both the Amplitude Envelope HMM and Time-Delay Embedded HMM are specified through an options structure that is passed with the data to the hmmmar.m function. This structure specifies a wide range of options including the number of states to infer, the type of observation model, the nature of the inference and some optional preprocessing. More details of the range of options that can be specified here can be found on the HMM-MAR wiki page^5^.

The hmmmar.m function computes the inference of the HMM parameters and returns several key variables. Firstly, the hmm struct is the HMM-MAR object containing a range of details about the state estimates, training options and priors. More details can be found here^6^. Secondly, the Gamma and vpath variables contain the inferred state time-courses. Gamma contains the full a-posteriori probability of each state at each time point, whilst the vpath contains the hard state assignment for each time point (known as the Viterbi path) following a Viterbi Decoding.

#### Amplitude Envelope HMM

The Amplitude Envelope (AE-HMM) was used as described in (Baker et al., 2014). The AE-HMM infers a multivariate Gaussian model on the amplitude envelopes of the source time-series. This section accompanies the code in hmm_2_envelope_estimation.m.

##### Data processing

The source time-courses were band-pass filtered between 2–40 Hz and symmetrically orthogonalised (see section “Parcellation and Leakage Reduction”) before the amplitude envelope was computed using the Hilbert Transform. The envelopes were then smoothed with a 100 ms moving average filter and normalized to have zero mean and standard deviation of one. Bad segments that had been set to zero prior to ICA were removed from the dataset and the continuous ‘good segments’ concatenated. The locations of the discontinuities between the good segments were passed to, and accounted for, in the HMM inference. This is important, in order to ensure that the HMM does not try to explain temporally separated data samples; for instance, the final sample in one good segment being used to predict the first sample of the next. Finally, the normalized envelope data were temporally concatenated across participants. The resulting input to the HMM is a matrix whose first dimension is the total number of ‘good’ samples across all participants after concatenation and the second dimension is the number of parcels.

##### Observation model

The observation model for each of the K states is a multivariate normal distribution defined across N nodes. An N×1 vector of mean values, and an N×N covariance matrix are specified.

#### Time Delay Embedded HMM

The Time Delay Embedded HMM (TDE-HMM) was used as described in (Vidaurre et al., 2017b). The TDE-HMM infers a multivariate Gaussian distribution describing a delay-embedding of the source time-courses. This section accompanies the code within hmm_3_embedded_estimation.m.

##### Data processing

The source time-courses were orthogonalised using Multivariate Symmetric orthogonalisation (see section “Parcellation and Leakage Reduction”). Bad segments were removed using the same procedure as the preprocessing for the AE-HMM. The sign ambiguity in the beamforming process means that data from the same parcel from different sessions may have arbitrarily opposite signs. Across a group-level dataset this can lead to suppression between group-level phase relations between nodes. To reduce this effect we applied the sign-flipping algorithm described in (Vidaurre et al., 2017b).

The source-reconstructed time-courses for each parcel were then time delay embedded using L lags. Here we set L to be 15, with values between -7 and 7. At 250 Hz this specifies a 30 ms lag in both directions. Increasing this window will increase sensitivity to lower rather than higher frequencies. The embedding creates a large NLxS matrix where S is the number of time samples in the dataset. The first dimension of this matrix containing the spatial and lag information was reduced by projecting the matrix onto the first 4N components of a PCA. This resulted in a final data matrix of size 4NxS, which was then used in the HMM inference.

Note that the number of PCA components retained across the time-delay embedding in the previous step affects the range of frequencies visible to the HMM. In general, fewer PCA components will bias the HMM towards lower frequencies due to the fact that lower frequencies tend to explain more variance in the data. A choice of 2N recommended as the minimum value, though in this case we chose 4N to ensure that the model can observe higher frequency content such as beta band dynamics (15–30 Hz).

##### Observation model

Similar to the AE-HMM, the observation model for each of the 6-states is defined as a multivariate normal distribution. This analysis is designed to emphasize the oscillatory signals within the MEG source space data, as such we only model the 4Nx4N covariance matrix within each state. The mean is not modeled in this case, as we expect oscillatory signals to be zero-mean after filtering and normalization.

#### Stochastic Inference

Once the data has been pre-processed and the observation model defined, the inference is the same for the AE- and TDE- HMMs, and makes use of the stochastic variational inference described in (Vidaurre et al., 2017a). The options for stochastic variational inference are selected by the variables starting with BIG in the hmmar options structure (e.g., options.BIG^∗^). Critically, the options.BIGinitbatch and options.BIGbatch specify the size of batches to use in the inference. This must be shorter than the number of elements in the T variable (i.e., the number of continuous data segments). As a general rule, making the batch size closer to numel(T) will make the inference slower and closer to the standard, non-stochastic variational estimation. In contrast, making the batch size smaller will make the inference faster and less memory intensive, but potentially noisier. Here we select a batch size of 15, meaning that the inference will consider 15 continuous segments at each iteration. A batch size of 10 or 5 is recommended for computers running on less than 32 Gb of RAM. The Stochastic Inference will try to use the parallel processing pool in MATLAB by default. On a very high performance computer, or for ease of debugging, this can be turned off by setting options.parallel = 0. This method is tractable for very large datasets and allows for a fully Bayesian estimation, providing full posterior distributions for each HMM parameter.

#### Run-to-Run Variability

It is crucial to ensure that the HMM results is stable across multiple runs of the inference. This step can be assess by running the HMM multiple times and qualitatively comparing the results of each iteration. A single run can be selected from amongst the alternatives by taking the inference with the lowest value of free-energy at the end of the inference. The free-energy is an approximation to the model evidence and as such, the model with the lowest free-energy can be taken as the one which best explains the data without becoming too complex. Here, the HMM inference was repeated ten times and analysis proceeds with the iteration with the lowest free-energy.

### HMM Global Temporal Statistics

Once the HMM has been inferred we can estimate a range of statistics reflecting the properties of the HMM states. The HMM inference returns a time course of posterior probabilities, Gamma, representing the probability that a state is on at each time point, and vpath, a Viterbi Path (Bishop, 2006) containing the mutually exclusive state allocations. Global statistics about the HMM dynamics were estimated from the Gamma time-course, as the posterior probabilities they represent are not mutually exclusive and are potentially more sensitive to cases where two or more states are approximately equally probable. First, the average life-time (also known as the dwell-time) of each state was computed as the average time elapsed between entering and exiting a state. Second, the fractional occupancy was computed across all time within a single participant’s dataset as the proportion of time spent in each state. Finally, the interval length was computed as the time elapsed between visits to a state. These metrics were computed in the same way for both variants of the HMM.

Example code can be found in the Temporal Statistics section of hmm_4_envelope_results.m and hmm_5_embedded_results.m, the computation uses the functions getFractional Occupancy.m, getStateLifeTimes.m and getStateInterval Times.m.

### HMM Validation

The HMM is highly sensitive to differences in variance; whether they arise from dynamics in underlying neuronal behaviour, biological artifacts (such as eye or head motion), acquisition artifacts (such as sensor jumps, periods of flat data or zeros), or differences between scan sessions (sensor noise or gain). Ideally, the HMM should only represent biologically relevant differences with a neuronal origin, so particular care must be taken during data pre-processing and normalizing. Artifacts or differences in noise that we might normally expect to “average-out” across trials or sessions may still lead to considerable distortion in the HMM inference; if a state is describing an artifact, it cannot contribute to the description of the neuronal dynamics.

The following practical checks are performed on the temporal statistics of the of the HMM to identify whether we are describing data artifacts or between session/subject variance:

1. States describing artifacts or session specific noise are likely to exhibit unusual temporal statistics. If the overall fractional occupancy or lifetimes for one or more states are very different to the other states, then they may describe artifacts. This can be confirmed by manual inspection of the raw time-series during periods for which the state is on.
2. Similarly, the temporal statistics for the states should be relatively consistent across sessions and participants. If a single session (or participant) is described by a single state, it is likely that a session specific difference in gain or noise is driving the state time-course. If this is the case, then we should check that the data are appropriately normalized within sessions prior to concatenation.

In practice, these checks should be completed once the HMM has been inferred on a new dataset, to identify any remaining artifacts which may then be removed and the HMM re-inferred. Once this process is complete, we can be confident that the HMM is focused on relevant dynamics rather than spurious noise sources. In the present dataset, these checks were used iterate tuning of the parameters used in the automatic bad-segment and ICA component rejection.

### HMM Task-Evoked Temporal Statistics

To see if the HMM state time-course, which has been inferred in an unsupervised manner with no knowledge of the task timings, shows task dependencies, we perform an analysis similar to an event-related potential/field on the Gamma time-course (i.e., the posterior probability of each state at each time-point). Specifically, the continuous time courses of the posterior probability, representing the probability that a state is on at each time point, were epoched around the presentation of the stimulus, and then averaged across trials within each participant. The resulting evoked fractional occupancy represents the proportion of trials in which the HMM was in a given state for each time-point within the epoch.

The evoked fractional occupancy was normalized by the baseline period (-130 to -30 ms before object onset), and the post-stimulus evoked fractional occupancy was passed into a two-level GLM. The first level GLM fits the evoked fractional occupancy across trials at each time-point for each participant using a trial-wise design matrix, and the second level computes the effect across participants at the group level, while modeling the between-subject variance as a random effect.

The first-level GLM has a design matrix with four regressors: a constant vector representing a mean-term and three regressors representing each trial-type by selecting the trials of each trial type from the famous face, unfamiliar face and scrambled faces conditions. The three condition regressors were demeaned prior to fitting the GLM. The parameter estimates for these predictors are summarized with three Contrasts Of Parameter Estimates (COPEs). One mean COPE and two differential contrasts are computed from the resulting parameter estimates. One contrast between faces and scrambled faces, and a second between the famous and unfamiliar faces.

The first-level estimates were carried forward to the group level, where the mean of each of the first level COPEs were fitted across participants with the first-level VARCOPEs included as a mixed-effects term. Statistical significance at the group-level was assessed using non-parametric permutations by sign-flipping (Nichols and Holmes, 2002; Winkler et al., 2014). One thousand permutations were computed with a maximum statistic taken across time and states to correct for multiple comparisons. A time-point was considered to be significantly different from baseline if its group level cope exceeded the 95^th^ percentile of the null distribution. These statistical procedures represent a test again the null hypothesis that the distribution of first level COPEs for each contrast has a zero mean. This is a standard approach for group-level statistics in neuroimaging (Winkler et al., 2014; O’Neill et al., 2015, 2017a). Other established statistical approaches could also be applied to the epoched Gamma time-courses, such as Fieldtrip’s timelock statistics^7^.

#### Spatial Maps and Connectivity

The spatial distribution of power or connectivity can be estimated directly from the fitted state-specific distributions in the observation model. For example, if we have a Multivariate Normal observation model fitting the mean and covariance of power envelopes, we may take the expectation of those distributions as the characteristic power and functional connectivity (in the form of power correlations). This provides an absolute value for each dimension of the observation model that highlights the features driving the inference. This method was applied to the results of the AE-HMM to generate state-wise mean activation maps directly from the values in the observation model. More specifically, the spatial maps from the AE-HMM are computed directly from the observation model by taking the expectation of the posterior distribution of the mean envelope value for each state and parcel. The inferred parameters of these distributions are contained within hmm.state. Example computation can be found in the Mean Activation Maps section of hmm_4_envelope_results.m.

We may also estimate a state’s characteristics *post hoc* by computing metrics across all time points when a particular state is being visited. This is more computationally intensive than direct description via the observation model, but allows for a wide range of state descriptors to be used beyond the information directly available in the observation model. This approach was used to estimate state-wise Power and Cross Spectral Densities from the results of the Embedded HMM. A state-wise multi-taper was used to estimate power and phase-locking coherence from the raw data weighted by each state’s posterior probability following the method in (Vidaurre et al., 2016, 2017b). This was repeated for each state and each parcel and was used as the basis of a whole-brain power map or Coherence (Nunez et al., 1997) network analysis.

The state-specific spectra are computed for the TDE-HMM analysis in the Statewise Spectra section of hmm_3_embedded_estimation.m. This makes use of the hmmspectramt function to compute the multitaper spectrum for each parcel and state separately for each participant. These are visualized in hmm_5_embedded_results.m.

#### Spectral Modes

The Power and Cross Spectral Density across the parcellation was computed from the spectral range of 1–40 Hz for each state. To aid visualization, Spectral modes are then computed by computing a Non-Negative Matrix Factorisation (NNMF) across the PSD estimates. The factorisation is carried out across all nodes, connections, states and participants. This was computed across 500 replicates of the Alternating Least Squares algorithm implemented in the MATLAB Statistics Toolbox (Berry et al., 2007). This is a data driven approach for spectral factorization and avoids setting arbitrary frequency bands of interest. The NNMF computes a low-rank approximation to the full solution. As this model is not unambiguously defined, the results can vary on repeated runs. Therefore, to ensure that the resulting spectral modes are interpretable as separate frequency bands, the NNMF was repeated twenty times an the results whose spectra was best approximated by single Gaussian distributions selected as the final result. This ensures that a factorisation with approximately unimodal spectra is used to visualize the HMM states. The four spectral modes approximately correspond to theta, alpha, beta and low gamma bands (see the central column in **Figure 5**). State-wise spatial maps and coherence networks were then estimated for each spectral factor and also thresholded for visualization using the GMM approach described above.

The NNMF is computed in the Spectral Mode NNMF section of hmm_5_embedded_results.m using the utils.run_nnmf.m function.

The NNMF is an optional step that avoids the specification of *a priori* frequency bands, however the user can choose to work within specific frequency bands if preferred. This is achieved by indexing into the frequency dimension in the psd or coh variables created by the multi-taper estimation in hmm_3_embedded_estimation.m. This approach is applied to generate the ‘broadband’ (1–30 Hz) network plots in **Figure 4**.

The phase-locking Coherence networks were thresholded for visualization using a Gaussian Mixture Model (GMM), as described in (Vidaurre et al., 2017b). This takes the distribution of all connection strengths, and models it as a mixture of two Gaussians, corresponding to one population of with typical connection strengths, and population with connection strengths that have unusually high values. Only the connections that are more probable to have been drawn from the Gaussian representing the high-valued population of connections (with the higher mean) are shown in the results. If the distribution is well described with a single Gaussian, we do not show any connections. This is implemented in the teh_graph_gmm_fit.m and used at the end of the Spectral Mode NNMF section of hmm_5_embedded_results.m.

## Results

### Amplitude Envelope HMM

The Amplitude Envelope HMM is inferred on the amplitude time-courses, and can identify states characterized as having distinct multi-region spatial patterns of amplitude and/or amplitude correlations.

#### Global Temporal Statistics

**FIGURE 3:**
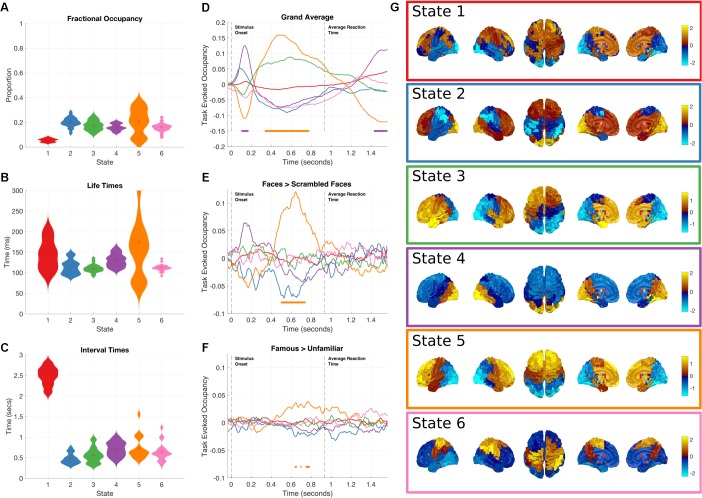
Results summary for the Amplitude Envelope HMM. The right column shows the overall temporal statistics estimated from the continuous data without considering task structure. The fractional occupancy **(A)**, Lifetimes **(B)** and Interval times **(C)** are shown. The middle column shows the group level results of the GLM analysis computed from the task-evoked fractional occupancies. **(D)** Shows the mean change in occupancy across all trials relative to baseline. Periods of significant change are indicated by a solid line at the bottom of the plot color-coded to state. **(E)** The result of the differential contrast between the Face and Scrambled Face stimuli. **(F)** The results of the differential contrast between the Famous and Unfamiliar face stimuli. **(G)** The mean activation maps for the six states extracted from the HMM observation models. The activation in each state is z-transformed.

The global temporal properties of the HMM are inspected through the state time-courses and posterior probabilities. The fractional occupancies, average lifetimes and interval times are summarized in **Figures 3A–C**. By inspection, we can see that the temporal properties of the states are relatively consistent. The average lifetime is around 100 ms and the mean interval is around 500–1000 ms, both consistent with previous literature (Baker et al., 2014). One exception is State 1, which has a much longer interval time of around 2.5 s, consistent with its lower occupancy.

Although all of the HMM inferences were blind to any knowledge of the task timings or structure in the data, the state dynamics may still have captured task relevant changes. These may be recovered by epoching the posterior probabilities of each state and averaging across trials for each time point in the epoch, to create the task-evoked fractional occupancy. Task-evoked analysis can then proceed on a state-by-state basis with the epoched posterior probability of each state. The results for the two-level GLM analysis of the trial-wise posterior probabilities of the HMM state time-courses can be seen in **Figures 3D–F**. The horizontal bars indicate periods of time which show significant increases in probability after sign-flipping permutations with maximum statistic multiple comparisons correction. All the states show some degree of modulation in their occupancy after the stimulus onset. We focus on the states whose occupancy increases. As the probability of each state must sum to one at any time point, an increase in the probability of one state will lead to a decrease in one or more of the others.

**Figure 3G** shows a spatial map of the mean amplitude envelope across all parcels in each state, this is taken as the expectation of the posterior distribution of envelope mean in the observation model. As with the previous application of the envelope HMM (Baker et al., 2014), these spatial maps reveal an interpretable set of networks. As the networks in this study come from data within a task context, they do not show an exact correspondence with the resting state networks as seen in (Baker et al., 2014). Instead, they reflect networks that are associated with the processing demands imposed by the visual decision-making task incorporating a motor response.

Next, we focus on individual states showing significant increases in occupancy relative to the pre-stimulus period.

##### Early occipital response

State 4 shows the earliest increase in task-evoked occupancy, peaking around 150 ms after stimulus onset with an increased occupancy of around 13% (**Figure 3D**). Neither of the condition contrasts between Faces and scrambled faces show a significant change in occupancy (**Figures 3E,F**) though the Faces>scrambled faces contrast shows a slight increase during the same time-window. This state is characterized by high values in the mean amplitude envelope across occipital cortex relative to the other states (**Figure 3G**). Given the timings and spatial distribution of the power of this state, it is likely to reflect the early visual processing of the stimuli.

##### Frontal response

State 5 has a sustained increase in occupancy during the latter part of the epoch, between 350 and 800 ms (**Figure 3D**). The increase reaches significance around 100 ms after the peak of state 4 and has dropped below threshold by the time of the average response at 932 ms. The contrast between Faces and Scrambled faces shows that the face stimuli have a larger occupancy than scrambled faces between 500 and 700 ms after stimulus onset (**Figure 3E**). In addition, the contrast between Famous and Unfamiliar faces reveals that famous faces lead to a greater occupancy in state 5 than unfamiliar faces during the same time-window as the previous contrasts, though the magnitude of the effect is much smaller. State 5 is characterized by high values in the mean envelope amplitudes in the frontal lobe relative to other states (**Figure 3G**).

##### Non-task responsive states

While not showing any significant changes in occupancy when locked to stimulus onset, the remaining states still characterize meaningful networks related to overall brain dynamics independent of the task of interest. State 2 is associated with high envelopes in the occipital and frontal lobes; and shows a small, non-significant increase in occupancy around 100 ms after stimulus onset. State 3 shows a left-hemisphere-lateralized fronto-temporal network whose task-evoked occupancy is similar to State 5.

### Time-Delay Embedded HMM

The Time Delay Embedded HMM is used on the raw time courses, rather than on the envelope time courses as is the case with the Amplitude Envelope HMM. Furthermore, it has the potential to identify states that have distinct multi-region spectra and/or phase locking networks.

#### Global Temporal Statistics

**FIGURE 4:**
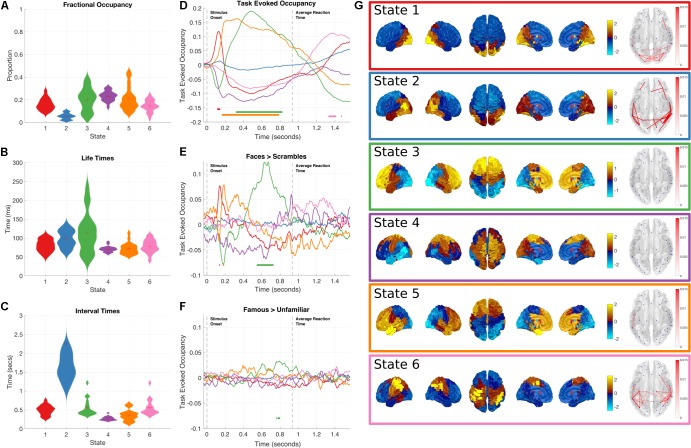
Results summary for the Time-Delay Embedded HMM, Note that these results are independently estimated from the results in **Figure 3**. The right column shows the overall temporal statistics estimated from the continuous data without considering task structure. The fractional occupancy **(A)**, Lifetimes **(B)** and Interval times **(C)** are shown. The middle column shows the group level results of the GLM analysis computed from the task-evoked fractional occupancies. **(D)** shows the mean change in occupancy across all trials relative to baseline. Periods of significant change are indicated by a solid line at the bottom of the plot color-coded to state. **(E)** the result of the differential contrast between the Face and Scrambled Face stimuli. **(F)** the results of the differential contrast between the Famous and Unfamiliar face stimuli. **(G)** The mean activation maps and Coherence networks for the six states extracted from the *post hoc* multi taper estimation. These results reflect wideband activation in each state and are z-scored across parcels.

As with the Amplitude Envelope HMM we can inspect the state time-courses to characterize their global dynamics (**Figures 4A–C**). All six states have similar fractional occupancies of around 15–20%, with state 2 occurring less than the other five states. The lifetimes of the Time Delay Embedded HMM states average between 50 and 100 ms, slightly faster than the Amplitude Envelope HMM. This is a reflection of the faster dynamics within the raw time-courses that are lost when computing the amplitude/power envelopes. The interval times average around 500 ms, with the exception of state 2 that takes around 1–2 s to reoccur, consistent with its lower global occupancy.

As with the Amplitude Envelope HMM, the state-wise task dynamics are recovered by epoching the posterior-probabilities of each state time-course and computing two first-level GLM fits, the first isolating the grand mean and the second computing contrasts between the Face and Scrambled face stimuli and the Famous and Unfamiliar Face stimuli. These results can be seen in (**Figures 4D–F**). In contrast to the Amplitude Envelope HMM, here we describe the states in full frequency resolution by computing the Power and Cross Spectral Densities using a multitaper on the raw data weighted by the posterior state probabilities. The wideband Power Spectral Density maps can be seen in **Figure 4G**.

Next we describe individual states that show significant task responses.

##### Early occipital response

State 1 has a significant increase in occupancy across trials around 150 ms after stimulus onset (**Figure 4D**), this increase is larger for the Faces than the Scrambled Faces stimuli (**Figure 4E**), but does not significantly differ for the Famous and Unfamiliar Faces (**Figure 4F**). The PSD shows that state 1 has relatively large broadband PSDs and coherences within the occipital lobes. This state is generally similar to State 4 in the envelope HMM, but we are now able to resolve coherence and a significant difference between the stimulus conditions.

##### Fronto-temporal response

Shortly after the occipital response, State 5 shows an increase in task-evoked occupancy. This increased probability is sustained from just before 200 to around 800 ms (**Figure 4D**), the increase is significantly larger for Faces than Scrambled Faces stimuli around 190 ms after stimulus onset (**Figure 4E**), this difference is very short-lived compared to the sustained increase in the mean across conditions, suggesting that the onset of State 5 might be earlier for Face stimuli. There is no significant difference between the Famous and Unfamiliar faces (**Figure 4F**). State 5 is characterized by a fronto-temporal power distribution with a peak in the Right Hemisphere temporal pole (**Figure 4G**). There is not an equivalent state in the envelope HMM, which only shows the occipital response within 200 ms of stimulus onset.

##### Frontal response

State 3 shows a sustained increase in task-evoked occupancy between 300 and 800 ms (**Figure 4D**). The occupancy of this state is larger for Face than Scrambled Face stimuli between 550–700 ms (**Figure 4E**) and briefly larger for Famous than Unfamiliar faces around 750–800 ms (**Figure 4F**). The power distribution of this state localizes it to within the frontal lobes. This state is similar to State 5 in the Amplitude Envelope HMM.

##### Post-movement motor response

Finally, State 6 has an increased occupancy starting from 1300 ms after stimulus onset (**Figure 4D**). Crucially, this occurs around 400 ms after the average response time from the button press, coinciding with the expected timing of the post-movement beta rebound. The power distributions of State 6 show power in left hemisphere motor cortex and right hemisphere parietal lobe (**Figure 4G**). This response shows no significant difference for either of the condition contrasts.

*Non-task responsive states:* State 2 and 4 do not show any significant changes in task-evoked occupancy (**Figure 4D**), or in either of the condition contrasts (**Figures 4E,F**). They correspond to a lateral occipital and a motor state respectively.

#### Frequency Resolved State Description

**FIGURE 5:**
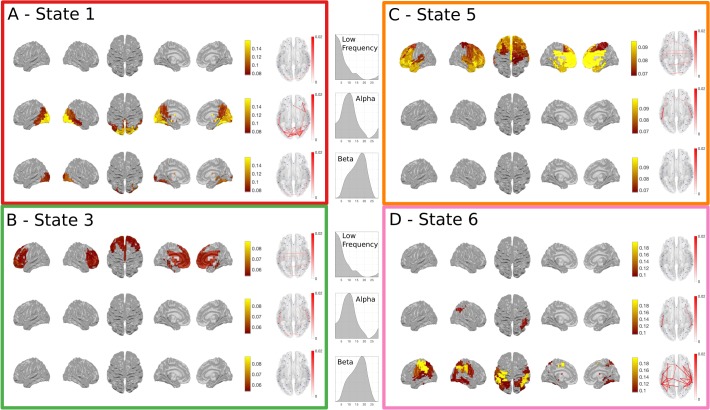
The spectrally resolved power maps and network coherence plots for the four task responsive states in the Time-Delay Embedded HMM. A shows the band-limited power for state 1, which showed an early increase in task-evoked occupancy. The top, middle and bottom rows show the power and connectivity for the low, alpha and beta bands respectively. **(B–D)** show the responses for the three other task responsive states with the same layout as **(A)**. **(B,C)** show that the majority of the power and connectivity in states 3 and 5 reside in the low frequencies in frontal cortex. Finally, **(D)** shows that the later responding motor state 6 is characterized by high power in the motor cortex.

We have only considered the wideband spectral content of the HMM states up to this point, yet the power correlations and coherence in MEG networks are known to differ across frequency (Hipp et al., 2012). Next, we breakdown the task responsive states to describe their frequency content as a complement to the results in **Figure 4**. A NNMF is used to identify modes within the power spectra across all participants and nodes. As this factorization is not uniquely identifiable, it was run several times until the resulting modes were unimodal to ensure that the results are readily interpretable, effectively imposing the prior assumption that we wanted to identify modes that were indeed unimodal. **Figure 5** gives a summary of the power and phase-locking of the task-responsive states from the Time-Delay Embedded HMM across the three identified NNMF modes, which broadly correspond to low-frequency, alpha and beta bands.

The power and coherence in State 1 reside almost exclusively in the alpha band (**Figure 5A**). In contrast, States 3 and 5 are characterized by low-frequency power and coherence in the frontal lobes (**Figures 5B,C**). Finally, the motor activation in State 6 is strongest within the beta band (**Figure 5D**).

#### HMM Reconstructed Time-Frequency Responses

**FIGURE 6:**
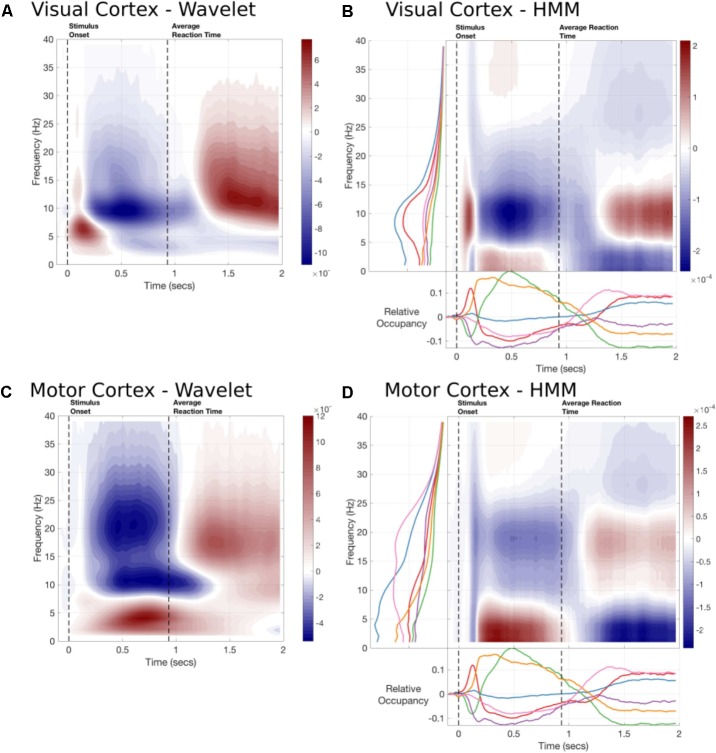
The time-frequency responses from two parcels estimated by Wavelet transform or constructed by the task-evoked state occupancies and state-wise power spectra. **(A)** A 5-cycle wavelet and the HMM constructed Time-frequency plot for the Occipital Pole parcel time-course. The state-wise spectra are shown in the left-hand subplot and the task-evoked occupancies in the bottom subplot. The overall time-frequency response is constructed from the outer product of these vectors summed across states. **(B)** A 5-cycle wavelet transform and HMM constructed Time-Frequency plot for a Motor cortex parcel.

As the Time Delay Embedded HMM provides us with a time-course and a spectrum for each state, we can use this to construct an alternative to task evoked standard time-frequency plots. **Figure 6** shows a summary of the TF response estimated by a 5-cycle wavelet transform and by the sum of the state-wise outer product of the task-evoked occupancy and the state PSD. The HMM-reconstructed TF response can be thought of as a regularized (or low-rank approximation) of the full TF plot and reflects the dynamics as represented by the HMM. Note that the task-evoked occupancy is the same for each parcel, the difference between parcels is carried by the spectrum, which is estimated for each parcel and state using a multi-taper.

The HMM-reconstructed TF responses are able to reconstruct the most prominent features of the wavelet plots. **Figure 6A** shows the response within the Occipital Pole. The HMM plot is able to characterize an early increase in alpha, followed by a desynchronisation and rebound around 1300 ms after stimulus onset (**Figure 6B**). This is primarily carried by the alpha power and early task-evoked occupancy change within State 1. In contrast, the response in parietal cortex does not show a strong evoked response (**Figure 6C**). Instead it is characterized by a more sustained increase in low-frequency or Theta power between stimulus onset and the button press followed by a rebound in Beta power. The HMM is able to reconstruct these patterns of activity using States 5, which is associated with low-frequency power in this parcel; and State 6, which shows a beta peak (**Figure 6D**).

## Discussion

Hidden Markov Models can describe the switching dynamics of large-scale brain networks on short, cognitively relevant time-scales. Here, we outline how a HMM can provide a framework for describing trial averaged induced power changes in task MEG data. Crucially, the HMM is able to describe dynamics arising from either sustained increases in oscillatory power or increase in the rate or amplitude of transient bursting activity. This rich description of rapid network dynamics in brain networks is tractable at both fast time-scales and across large-scale brain networks. The present results show that rapid switching between large brain networks estimated without knowledge of any task structure can carry pertinent task structure and provide a rich description of how the brain solves a task by dynamic reorganization of large-scale brain networks. The AE-HMM and TDE-HMMs yield broadly consistent results, though the TDE-HMM – working on the raw time-series rather than collapsing by broad-band enveloping – is directly sensitive to spectral content and is able to resolve a richer description of the task structure at higher temporal resolution including phase relationships among multiple regions.

### Statistical Assessment

Statistical testing of dynamic networks can lead to combinatorial explosion of tests across connections, time and experimental conditions. In electrophysiological data, frequency often becomes an essential additional dimension. This leads to both difficulty in establishing and summarizing the most salient effects in the data and ensuring that multiple-comparisons correction is carried out appropriately. The HMM aids in both of these issues. The state observation models naturally provide high-level summaries of the data and statistics can be performed on a limited number of state time-courses rather than across individual parcels, connections and frequencies. As such, the HMM is able to describe complex, dynamic networks in a tractable, interpretable and statistically both valid and efficient way.

### Transient States and Bursting Oscillations

The task-evoked structure within this paper arises from epoching the HMM state time-courses after inferring state time-courses without knowledge of task events or timings. The resulting task-evoked occupancies are smooth and often imply sustained changes in occupancy throughout the task epoch, yet they are constructed from transient states that are discrete at the single trial level. For instance, state 5 in the TDE-HMM shows a sustained increase in task-evoked fractional occupancy lasting around 600 ms starting from 175 ms after stimulus onset. Yet, the average lifetime of each state visit for state 5 is around 80 ms. This result is in line with recent work which suggests that task-evoked time-frequency responses occur from brief bursts of oscillation which only appear to be sustained once we average across trials (Shin et al., 2017). This bursting perspective suggests that some neuronal oscillations arise from transient events that may be better characterized by their rate or duration rather than absolute power. Indeed, such parameters are cognitively relevant across a range of data modalities (Shin et al., 2017). In addition, deep brain stimulation selectively targeted at transient bursts in beta power outperforms tonic stimulation in reducing motor impairment in Parkinson’s Disease (Tinkhauser et al., 2017). In that respect, HMM – by its discrete nature on single-trial level - might help to uncover mechanisms such as oscillatory bursting that so far have been buried in the averages performed by conventional time-frequency analysis.

### HMM in Relation to Sliding Windows and ICA

Hidden Markov Modeling addresses some of the limitations of sliding window approaches for estimating dynamic functional connectivity (O’Neill et al., 2017a). Whilst sliding window methods estimate connectivity within short, uniform data segments spanning the continuous time-series, the HMM decomposes the data efficiently and unsupervised by inferring adaptive data segments for each state and estimating the connectivity across all visits to that state. This removes the necessity of pre-specifying window length and the need for windows to be sufficiently long to robustly estimate a large-scale functional connectome. Instead, the features of these windows (such as distribution of life-times etc.) become interesting properties in themselves; they are accessible to analysis and might carry functional significance.

While we are proposing that the HMM helps to overcome some of the limitations of sliding window approaches, clearly sliding-window approaches are still important and useful. This includes when working at slow time-scales, for which the requirement of using longer windows is not prohibitive; and when working with metrics of functional connectivity that cannot be straightforwardly represented as a generative model in the HMM.

Another alternative approach to network dynamics is temporal ICA estimation, which identifies components within a dataset based on their temporal independence. A shortcoming of ICA is that standard ICA components are based exclusively on spatial features. HMM states, instead, are probability distributions that can capture rich spectral properties in the data, including information of power and phase. This is the case of the embedded HMM and the HMM-MAR. The HMM thus provides a more powerful approach for characterizing spectrally-defined networks in electrophysiological data.

### Limitations

The choice of parcellation is an important preprocessing stage; parcellation is a spatial dimensionality reduction that makes the HMM network modeling more tractable and robust to small spatial variations across participants. The choice of atlas is crucial; it defines the sampling across space and will ideally reflect the effective resolution of the underlying source solution. Here, we have used a cortical parcellation from (Colclough et al., 2015). This 39 region parcellation has been previously used to reliably estimate large-scale static functional connectivity networks in MEG (Colclough et al., 2017, 2016). The use of such a relatively coarse parcellation is consistent with evidence that the effective dimensionality in MEG source space (following Maxfiltering) is approximately 64 (Taulu and Simola, 2006), and with the findings from an adaptive parcellation approach (Farahibozorg et al., 2018). Nonetheless, exploring the definition of ‘optimal’ functional parcellations is an active field of research in both fMRI and MEG (Glasser et al., 2016; Farahibozorg et al., 2018). Note that the pre-processing code can easily make use of a different parcellation by inputting a different nifti file into the ROInets.get_node_tcs call in hmm_1_preprocessing.m. However, it should be noted that the number of parcels should be less than the rank of the data (which in our case was ∼60 following Maxfilter and ICA denoising) in order for the spatial leakage correction to work, and which is clearly a sensible constraint to apply regardless.

The HMM makes a number of assumptions to ensure that the inference is tractable. Firstly, we assume a fixed number of states (K). The objective is not to establish the ‘correct’ number of states, but to identify a number that provides a description of the dataset at a useful granularity. This is analogous to the choice of the number of components in an ICA decomposition. Nonetheless, as in ICA, care must be taken to ensure that the results are reasonably robust to the choice of K. Here we followed the approach in (Baker et al., 2014) who explored the network maps from a range of choices of K. Increasing K above 6 did not change the topologies of the most prominent states or their task profiles. For simplicity in this tutorial paper, this test was performed on the envelope HMM and applied to both the AE-HMM and TDE-HMM.

Another core assumption in the HMM is that the states are mutually exclusive, in that only a single state may be active at a single time-point. This may be potentially undesirable in descriptions of brain dynamics in which multiple networks are often though to operate in parallel. However, the Bayesian implementation of the HMM inference used here provides the posterior probabilities of each state at each time point are also returned in the variable Gamma. These posterior probabilities are not mutually exclusive and may identify times in which two or more states are equally probable. Further, it is worth noting that network multiplexing can also be realized at slower time scales, for example, through temporal correlation of the rate of occurrence of state fractional occupancies at slower time scales. Addressing the information contained in the state time courses at multiple time scales is an important area for future investigations.

### Future Work With HMMs

The HMMs presented in this paper are designed to explore whole brain states during continuous data recordings that are then interrogated to explore state-wise task dynamics. As such, the results focus on the large-scale trends in power and functional connectivity during the task leading to networks describing effects such as an early alpha response in occipital cortex or a post-movement beta rebound in motor cortex. More subtle effects within the data could be probed with hypothesis driven design choices when setting up the HMM. For instance, the HMM could be inferred on a parcellation restricted to occipital cortex to further explore the states involved in visual processing. Similarly, the HMM inference could be restricted in time to infer states only from epochs of interest within the task.

We may also adapt the constraints on the HMMs observation model or state time-courses to explore a specific question. These analyses may require additional code and analysis steps not detailed here, but are potentially of interest to a range of cognitive and clinical applications. For example, a HMM may be inferred on one dataset to identify a set of observation models defining states. These states can then be fixed and state-time courses inferred from a second dataset. Another alternative is to constrain the order of state visits and only allow the duration to vary. This approach was applied in (Baldassano et al., 2017) to identify individual variance in event structure when viewing or recalling video sequences. Finally, the HMM inferred in this manuscript is unsupervised with respect to task and condition structure, however the inference may be tuned to perform supervised learning. We may infer a HMM whose states allow the maximum decoding of task conditions or behavioral performance.

To summarize, we have presented a pipeline for the analysis of rapid dynamics in large-scale brain networks using HMMs. The HMM is a flexible framework for describing the induced power changes in electrophysiological datasets and provides insight into how smooth trial-averaged responses can be constructed from transient bursting events. The utility of the HMM framework in task data is demonstrated in the very rapid task-evoked and condition sensitive changes in fractional occupancy of the states; representing dynamic functional connectivity on cognitive time-scales.

## Ethics Statement

This study makes use of existing neuroimaging data which is freely available online. The data set is published online, with details about the associated ethical procedures here: Wakeman and Henson (2015).

## Author Contributions

AQ and MW designed the study. AQ and RA carried out the data analysis and organized the scripts for sharing. AQ, DV, RA, RB, AN, and MW wrote the manuscript.

## Conflict of Interest Statement

The authors declare that the research was conducted in the absence of any commercial or financial relationships that could be construed as a potential conflict of interest.

## Footnotes

1 https://ohba-analysis.github.io/osl-docs/

2 https://github.com/OHBA-analysis/HMM-MAR

3 https://osf.io/ugjbr/

4 https://openfmri.org/dataset/ds000117/

5 https://github.com/OHBA-analysis/HMM-MAR/wiki/User-Guide#-hmm-mar-model-estimation

6 https://github.com/OHBA-analysis/HMM-MAR/wiki/User-Guide#struct

7 http://www.fieldtriptoolbox.org/development/cluster_permutation_timelock
